# Synovial fluid level of myeloperoxidase enzyme following ultrasound-guided regenerative injections in patients with irreducible temporomandibular disc displacement: a double-blinded parallel randomized clinical trial

**DOI:** 10.1186/s12903-026-09266-7

**Published:** 2026-07-20

**Authors:** Mariam M. Bahgat, Nadia Ragheb El-Helw, Ahmed M. Abdel-Hamid, Mohamed Mohamed Fata, Medhat Saber Ashour, Aly Mohamed Atteya

**Affiliations:** 1 Lecturer of Prosthodontics, Faculty of Dentistry, Alexandria University, Alexandria, Egypt; 2 Faculty of Dentistry, Alexandria University, Alexandria, Egypt; 3 Professor of Maxillofacial and Plastic Surgery, Faculty of Dentistry, Alexandria University, Alexandria, Egypt; 4 Professor of Microbiology, High Institute of Public Health, Alexandria University, Alexandria, Egypt; 5 Associate Professor of Maxillofacial and Plastic Surgery, Faculty of Dentistry, Alexandria University, Alexandria, Egypt

**Keywords:** Growth factors, Limited mouth opening, Myeloperoxidase enzyme, Prolotherapy, Regenerative injection, Temporomandibular disorders

## Abstract

**Background:**

Irreducible temporomandibular disc displacement (DDwoR) is a subtype of internal derangement of temporomandibular joint (TMJ-ID). Recognizing the risks related to DDwoR with its pathogenesis, the treatment of this disorder becomes essential. Prolotherapy, also known as regenerative injection therapy, has been emerging as one of the most promising approaches to treat patients with TMJ-ID. The current prospective parallel randomized clinical trial (RCT) was done to assess the effect of hypertonic dextrose (HD) regenerative injection and that of concentrated growth factors (CGF) in conjunction with a stabilization occlusal appliance (SA) in the management of DDwoR.

**Methods:**

Twenty-four patients suffering from DDwoR were randomly assigned to 2 equal groups; Group I (HD + SA), Group II (CGF + SA). Assessments were done before treatment, 1 week, 4 weeks, and 24 weeks after the treatment using the Visual Analogue Scale of Pain (VAS) and assisted maximum mouth opening (MMO). Moreover, synovial fluid (SF) samples were obtained for the analysis of the Myeloperoxidase enzyme (MPO) levels, before and 6 months after treatment.

**Results:**

A significant relief in pain was found in both groups at 1 month and lasted up to 6 months postoperatively (*p* < 0.001). Moreover, assisted MMO in both groups was significantly increased (*p* < 0.001). As for MPO levels in SF, by 6-months from the intra-articular injection, the enzyme levels were significantly reduced in both groups (*p* = 0.002). Although the MPO concentrations in the SF were significantly higher than that found after injection, there was a statistically insignificant difference between groups.

**Conclusions:**

We concluded that injection of HD or CGF in the superior joint space could be a valuable treatment option for patients suffering from DDwoR. As both led to a positive impact on MMO, and the relief of joint pain with the decrease in levels of inflammatory biomarker inside the joint.

**Trial registration:**

The current trial is a prospective assessor and analyst blinded RCT that has been registered at ClinicalTrials.gov with identification number: NCT04557878, 1/4/2020.

## Background

Irreducible temporomandibular disc displacement (DDwoR) is one of the subtypes of TMJ-ID, where the articular disc is anteriorly displaced to the condylar head in closed and open-mouth positions resulting in interference in the mandibular movements. TMJ pain as well as limitation in mandibular range of motion are common signs and symptoms associated with DDwoR [1, 2].

Compromised retro-discal tissue is believed to be one of the factors that lead to disc displacement as it has a key role in holding the articular disc in its normal position [3, 4]. In addition, for the disc to shift over the condylar head, a deformation of its structure together with damage to the discal ligaments may occur. Strengthening these ligaments, fibro-osseous junction, and repair of the disc structure where collagen and elastin play a role in restoring the disc shape and position could help in improving the disc position which results in restoring range of mandibular motion.

Another factor that plays a role in the pathogenesis of DDwoR is the composition of the SF of TMJ. Inflammation inside the joint affects the viscosity of the SF leading to insufficient lubrication and nourishment of TMJ. One of the inflammatory mediators detected in the SF of joints with TMJ-ID is MPO. MPO activity observed in the SF of these joints is an indication of neutrophil infiltration into the tissue and increased tissue damage which may lead to osteoarthritis [5–7]. The goals for treating patients with DDwoR should not only be the relief of TMJ pain and dysfunction but also the prevention of disease aggravation.

Recognizing the risks related to DDwoR with its pathogenesis, the treatment of this disorder becomes essential. Conservative as well as minimally invasive treatment modalities including physical therapy, biobehavioral therapy, occlusal appliances (OAs), pharmacological therapy, intra-articular injections or combination of these treatments should be considered prior to any surgical interventions [8–14].

Among the various designs of OAs, hard stabilization appliance (SA) is found to be effective in treating joint pain associated with TMDs making an essential auxiliary treatment [15, 16]. Furthermore, several methods [17–19] have been suggested for the fabrication of SA including conventional methods to the use of computer aided designed and manufactured (CAD/CAM) ones.

As for the minimally invasive intra-articular injections in the TMJ, lavage of the joint cavity, viscosupplementation of hyaluronic acid and corticosteroids are commonly known and used [20–22]. However, in recent years, prolotherapy or regenerative injection therapy, has emerged as one of the most promising approaches to treat patients with TMDs in which the word ‘‘Proles’’ means growth [23, 24]. Prolotherapy injections could be an injection of growth factors or an injection of a liquid that induces the body to produce growth factors; like hypertonic dextrose injection (HD) with concentration ranging above 10%. When dextrose is injected in painful joints, ligaments and or adjacent joint spaces, it will result in an inflammatory response in the local injected tissues, which in turn will trigger the release of growth factors as connective tissue growth factor and fibroblast growth factor. This promotes healing in the form of structural as well as functional integrity resulting in reduction in pain and improvement of function [25, 26]. The effect of HDPT on ligaments, tendons, and cartilage is well documented in the literature; in which concentrations above 10% have been reported to have inflammatory as well as regenerative effects resulting in the formation of new collagen fibrils promoting healing of injured tissues. On the contrary, concentrations below 10% have an anti-inflammatory effect. Although there is no consensus on the concentration of HD for the treatment of ID, concentrations from 10% to 50% have been reported in literature [23–26]. These concentrations proved to be effective in the relief of TMJ pain and improvement in the MMO both in the short and long term. However, the most common concentration used in those studies was 12.5% HD. Furthermore, the recommended treatment protocol for HD is using multiple injections. These multiple injections are needed to achieve a proliferative effect as these repeated injections ensure a sustained inflammatory response that is crucial for stimulating the repair process of the injured tissues [23–26]. 

Another form of regenerative injections that has gained attention as TMJ intra-articular injection is the injection of autologous blood or its derivatives as Platelet concentrates (PC) including platelet-rich plasma (PRP), platelet-rich fibrin (PRF), and concentrated growth factors (CGF) [27–34]. The hypothesis of PC is related to in situ release of autogenous growth factors such as platelet-derived growth factor, transforming growth factor, vascular endothelial growth factor, insulin-like growth factor, fibroblast growth factor, and epidermal growth factor with anti-inflammatory effects and healing enhancing properties; hence, stimulate cell proliferation and tissue repair which promote a revolution in regenerative medicine. There are significant differences in the preparations protocols of PRP, PRF, and CGF resulting in significant differences in amounts of growth factors (GFs) produced. PRF and CGF produce significantly more GFs as compared to PRP [27–33]. 

Liquid phase concentrated growth factor (LPCGF), the latest generation of PC, was first described by Sacco [31], and produced by the centrifugation of venous blood with no additive using alternating centrifugation speeds for 12 min. Over recent years, several studies [35–37] have described the application of PC through intra-articular injection for curing TMDs. However, to our knowledge, no randomized clinical trials (RCT) were found in the literature comparing the intra-articular injection of HD to that of CGF in the management of DDwoR. Therefore, it was of essence to investigate the effectiveness of such treatment protocols on a clinical level, as well as on a molecular level. Our null hypothesis was that there is no statistically significant difference between the two treatment protocols.

## Materials and methods

### Trial design

This study is a parallel prospective RCT that was registered at ClinicalTrials.gov with identification number NCT04557878 and has been granted ethical approval from the Research Ethics Committee of the Faculty of Dentistry, Alexandria University, Egypt (international number: IORG0008839, committee number: 0128-04/2020; registration date: 1/4/2020). Patients were enrolled in the present clinical trial following the signing of a written informed consent form.

### Sample size calculation

The G*Power software program version 3.1.9.6 (Heinrich-Heine-Universität Düsseldorf, Germany) was used to calculate the sample size of 24 patients with a 0.05 significance level **α** error and 80% power ^28^. This sample size was calculated according to data from previous studies [24, 37] assessing the effect on the MMO as it was our primary outcome. The mean ± SD difference in MMO after 6 months for patients treated with HDPT was 1.43 ± 4.37 [24] while the mean ± SD when LPCGFs was used was 9.59 ± 7.32 [37]. The sample size was calculated to be 10 patients per group which was increased to 12 to make up for loss to follow up.

### Inclusion and exclusion criteria

**Fig. 1:**
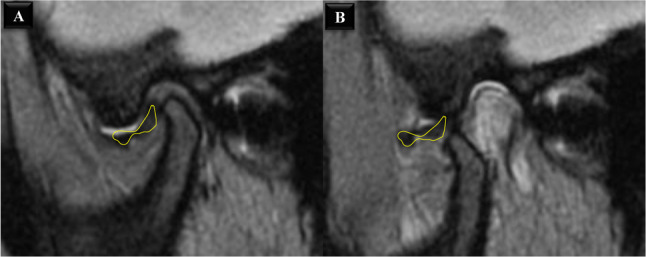
MRI showing DDwoR: (**A**) MRI in closed mouth position showing articular disc displaced anteriorly (outlined in yellow), (**B**) MRI in closed mouth position showing that the articular disc is still anteriorly displaced confirming DDwoR (outlined in yellow)

The present RCT was performed in Temporomandibular Disorders Clinic at the Prosthodontic Department, Faculty of Dentistry, Alexandria University. All patients enrolled had pain in the TMJ region. The patient-related selection criteria were limitation in assisted maximum mouth opening (MMO < 40 mm); irreducible anterior disc displacement detected by magnetic resonance imaging (MRI) (Fig. 1); age ranges between 20 and 40 years old; presence of full set of natural teeth; Patients with angle class I occlusion; retruded cuspal position not more than 2 mm and with no open bite. Subjects were excluded if they had: reducible disc displacement shown in MRI; inability to undergo MRI; neurologic or hematologic disorders; inflammatory diseases; uncontrolled systemic disease; ongoing anticoagulant drug therapy.

According to the above-mentioned strict selection criteria, subjects were selected and clinically examined through Orbach’s Diagnostic Criteria for Temporomandibular Disorders (DC/TMDs) [38] by an experienced blinded investigator. 24 patients diagnosed with DDwoR were enrolled in the current RCT.

### Randomization

Patients enrolled in the present trial were assigned into one of the study groups randomly. A serial of number was assigned to each patient which will be used in the allocation. A copy of these numbers with the patients’ respective names was noted and enfolded in opaque sealed envelopes. A trial independent interpreter randomly allocated the patients to 2 equal groups; HD + SA group (I), and CGF + SA group (II) using computer-generated list of random numbers. Specific software (Sealed Envelope, UK) was used to create randomization sequence in blocks of 2. Then, the allocated group was written down and the paper was put in an opaque envelope with the patient’s number, then the envelope was sealed. At the intervention time, a trial independent assistant unsealed the envelopes, and indicated the group to which the patient was assigned.

### Intervention

**Fig. 2:**
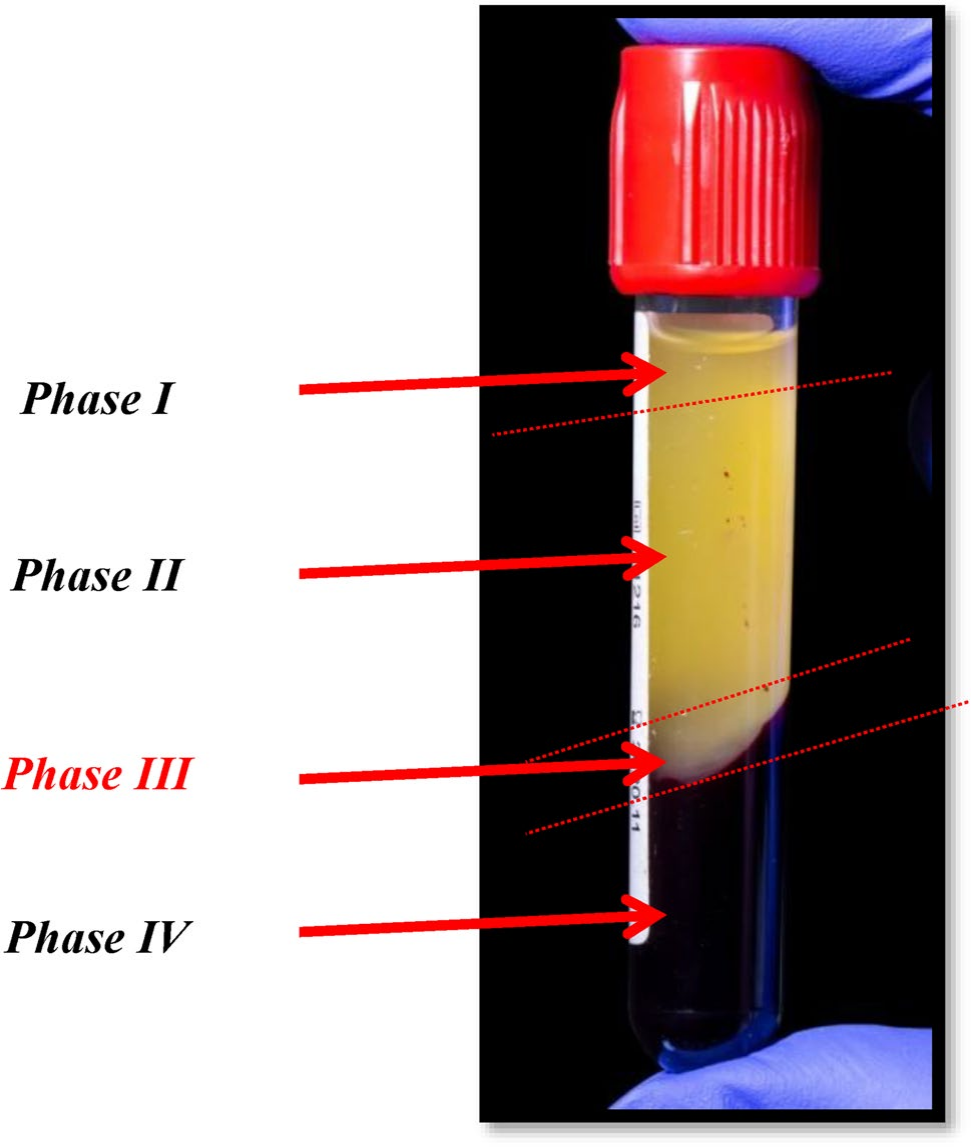
Blood after centrifugation (phase I: Platelet Poor Plasma; phase II: Buffy Coat; phase III: LPCGF; phase IV: RBCs )

Patients in group I received an injection of 2 mL of 12.5% HD solution (Otsuka Pharmaceutical Co.) in the superior joint space, under local anesthesia once; and repeated every month for 2 consecutive months if needed [26], in conjunction with a CAD/CAM full–arch hard clear SA on the maxillary arch. Patients in group II received, under local anesthesia, a single intra-articular injection of 2 mL of CGF in conjunction with a CAD/CAM full–arch hard clear SA on the maxillary arch. The CGF was prepared from 10 ml the patient’s venous blood collected in vacutainers without the addition of anticoagulants, then were immediately centrifuged in a medical centrifuge that is designed to be preprogrammed on the recommended parameters by Sacco et al. [31] at 947 RCF for 2 min, 748 RCF for 4 min, 947 RCF for 4 min, 1169 RCF for 3 min, deceleration for 36s then stop. By the end of this process, blood fractions were formed (Fig. 2). The LPCGF layer was then aspirated from vacutainers to be injected in the superior joint space. This process was done by one operator.

### Intra-articular injection procedure

**Fig. 3:**
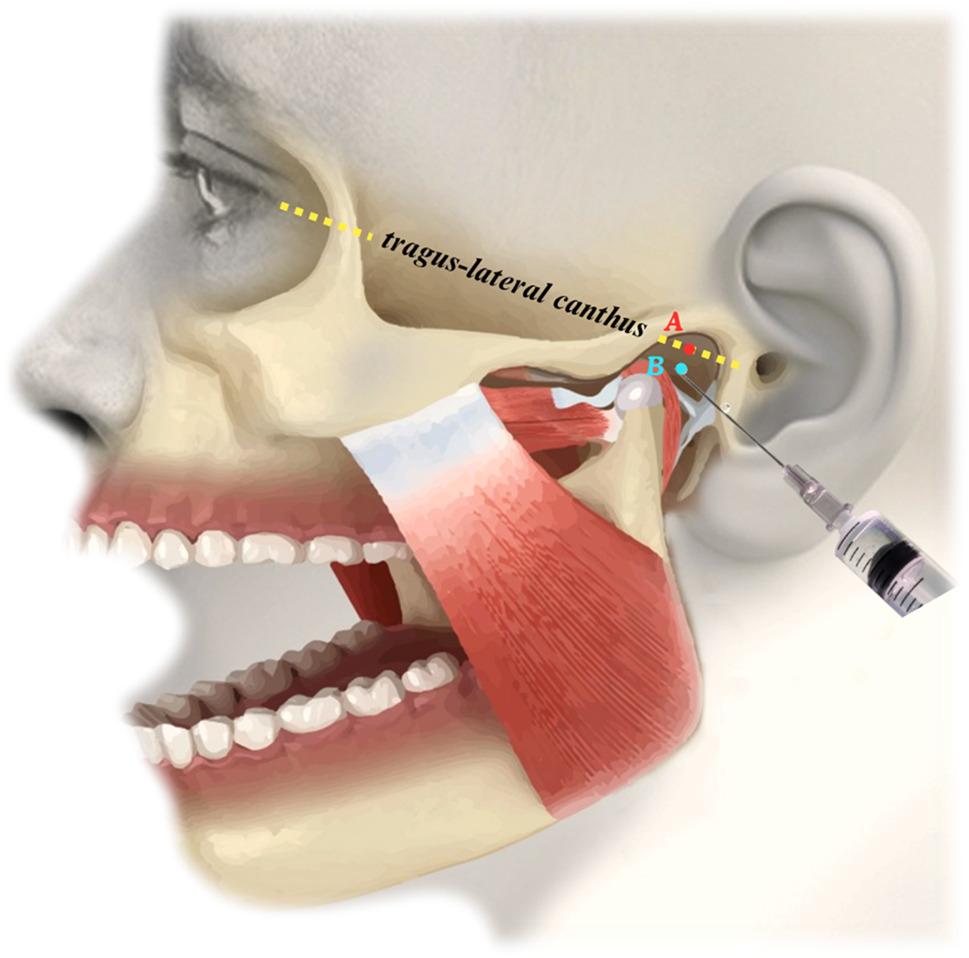
Illustrative diagram of intra-articular injection technique. Point of needle entry (B) is 2 mm below point (A), 1 cm from tragus

After auriculotemporal nerve block injection was given, SF was collected from the superior joint space then ultrasound- guided (US-guided) injection procedure was done following Murakami method [39] (Fig. 3).

### Stabilization appliance CAD/CAM technique

Intraoral scans of both arches were obtained by an intraoral scanner (Cerec omnicam, Dentsply Sirona). Then an anterior deprogrammer was stabilized on the maxillary central incisors using putty condensation silicone material (Zetaplus, Zhermack) with the incisal plateau perpendicular on the long axes of the mandibular central incisors; then musculoskeletal stable position was determined using bilateral manipulation technique. A buccal scan was taken at the musculoskeletal stable position, and the scanned data was then exported as Polygon File Format.

Then these files were imported into the CAD software (DentalCAD, exocad, GmbH) to design the SA on the maxillary arch. The finished design of SA was then saved as a standard tessellation language file.

**Fig. 4:**
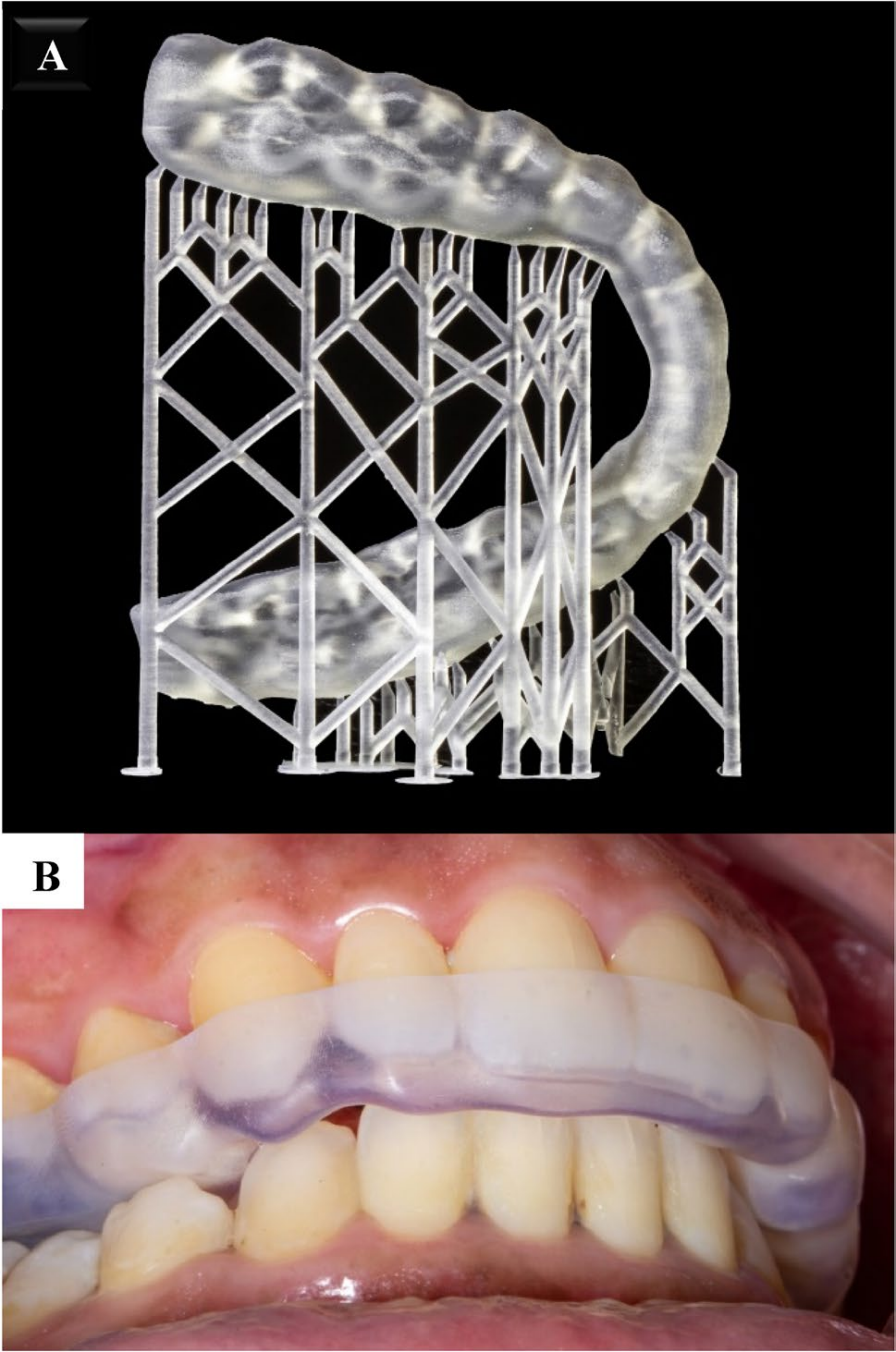
Showing stabilization appliance: **A**, 3D printed SA, **B**. Intraoral View of SA in place

The appliance was then printed from biocompatible clear resin using a desktop 3D printer (Form 2, Formlabs) (Fig. 4A). All patients were informed to use the appliance at night (Fig. 4B).

### Assessment

#### Clinical evaluation

Level of TMJ pain was assessed using Visual analogue Scale of Pain *(VAS*) from 0,representing no pain, to 10, indicating the worst pain. In addition, assisted maximum mouth opening (*MMO*) was recorded. All clinical assessments were done before any treatment as well as after 1 week, 4 weeks, and 24 weeks from treatment.

#### Biochemical analysis

The SF collected from the superior joint space was analyzed using Simplestep enzyme-linked immunosorbent assay (ab272101 Human MPO SimpleStep ELISA Kit, Abcam) to determine the MPO enzyme activity following the manufacture’s protocol. The level of MPO enzyme was measured pretreatment and 6 months posttreatment.

#### Blinding

Due to the nature of the intervention, it was impossible to have the operator and the patients blinded during the procedure. However, both the outcome investigator who did the follow-up evaluation and the analyst were blinded to the study group the patient was in; hence, it was a double-blinded clinical study.

### Statistical analysis

After gathering all data, it was inserted into a personal computer and SPSS software packages, version 20 (Armonk, NY: IBM Corp), was used to perform statistical analysis [40].

To validate the normality of distribution of the data, the Shapiro-Wilk test was applied. Parametric tests including student t-test, and ANOVA with repeated measures test were used for normally distributed quantitative data, while non-parametric tests including Mann-Whitney, Wilcoxon signed ranks, and Friedman tests were applied for abnormally distributed quantitative variables. Furthermore, Spearman’s rank correlation was performed to assess the relation between MPO and both MMO and VAS. The level of significance was set at 5%.

## Results

### Subjects enrolled

**Fig. 5:**
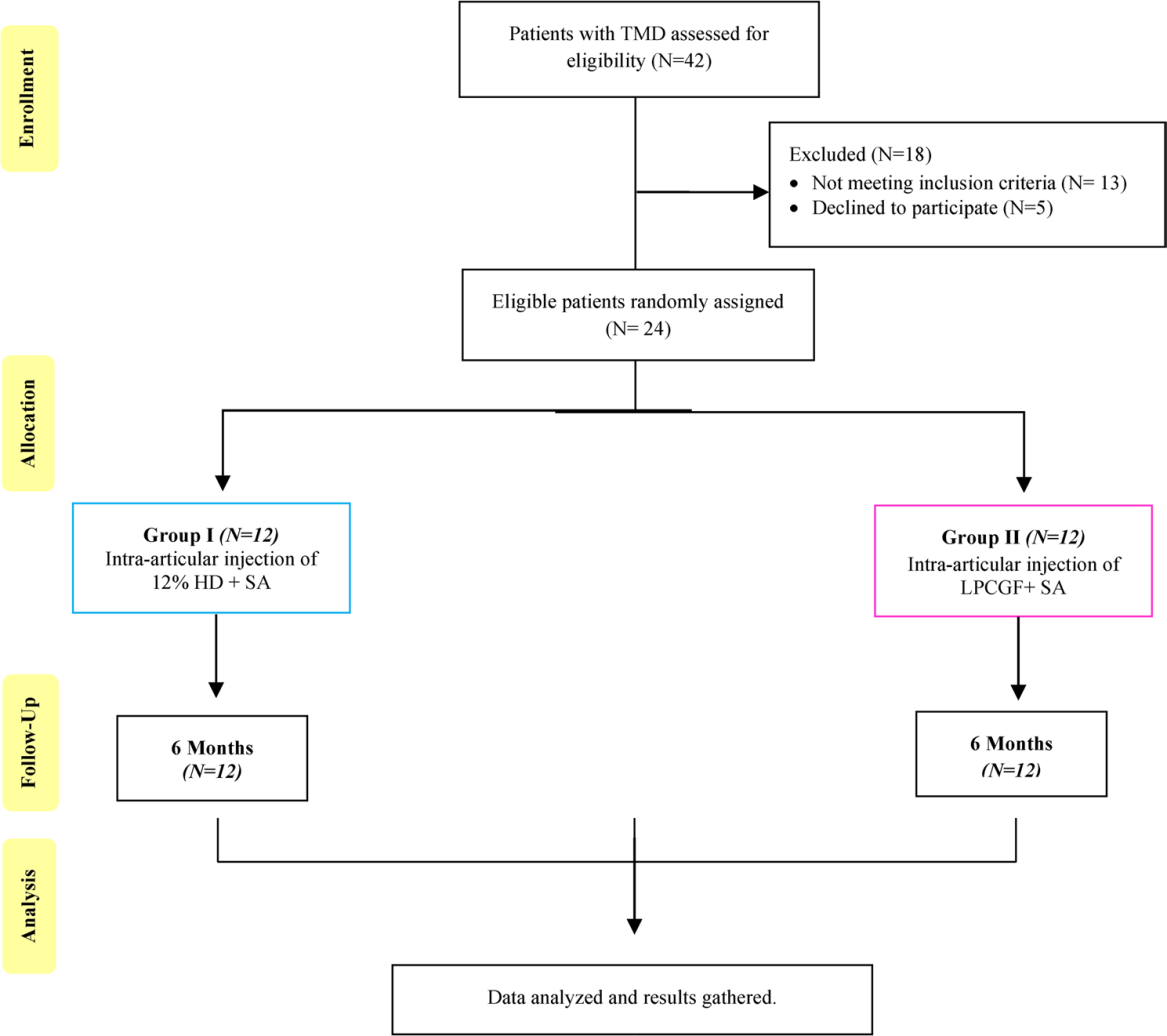
CONSORT Flow chart of the study. TMD, Temporomandibular disorder; N, number of patients; HD, Hypertonic dextrose; SA, Stabilization Appliance; LPCGF, Liquid phase concentrated growth factors

Primarily, the socio demographic data of subjects were collected, and a statistically insignificant difference was observed (Table 1). Based on the strictly identified selection criteria, 24 patients were enrolled in the current clinical trial. At the follow-up periods, the data were gathered then analyzed statistically as presented in the CONSORT flowchart (Fig. 5). All subjects completed the trial with no adverse effects noted.

**Table 1:** Sociodemographic data of the subjects and TMD symptom period

	Group I *(* *n* * = 12)*	Group II *(* *n* * = 12)*	*p*
Gender			
Male	0 (0.0%)	0 (0.0%)	–
Female	12 (100.0%)	12 (100.0%)	
Age (years)			
Min. – Max.	20.0–30.0	20.0–32.0	0.664
Mean ± SD.	25.33 ± 3.89	24.58 ± 4.44	
Marital Status			
Single	2 (16.7%)	2 (16.7%)	^FE^p= 1.000
Married	10 (83.3%)	10 (83.3%)	
Education			
College	11 (91.7%)	10 (83.3%)	^FE^p= 1.000
High	1 (8.3%)	2 (16.7%)	
Working Status			
Student	2 (16.7%)	4 (33.3%)	0.453
Working	5 (41.7%)	2 (16.7%)	
Housewife	5 (41.7%)	6 (50.0%)	
TMD symptoms period (months)			
Min. – Max.	12.0–60.0	12.0–60.0	0.713
Median (IQR)	24.0 (18.0–48.0)	24.0 (12.0–48.0)	

Group I, HP + SA; Group II, CGF + SA; SD, Standard deviation; p, difference between the two groups; FE, Fisher Exact test; IQR, Inter quartile range

### Clinical evaluation

#### Visual Analogue Scale of pain (VAS)

For TMJ pain assessed by VAS, a significant relief in pain was found in both groups at 1 month and lasted up to 6 months postoperatively (*p* < 0.001). However, no significant difference was found between the study groups through all the follow-up periods (*p* > 0.05) (Table 2).

**Table 2:** Comparison between the different studied periods according to Visual analogue scale of pain (VAS) in each group

VAS	Pre	1week	1month	3months	6months	*p*
Group I *(n = 12)*						
Min. – Max.	7.0–10.0	1.0–5.0	0.0–4.0	0.0–2.0	0.0–2.0	< 0.001^*^
Mean ± SD.	8.50 ± 1.09	3.0 ± 1.35	1.25 ± 1.86	0.33 ± 0.78	0.17 ± 0.58	
* p*_*0*_		*0.081*	*< 0.001* ^*^	*< 0.001* ^*^	*< 0.001* ^*^	
* p*_*1*_			*0.028* ^*^	*0.002* ^*^	*0.001* ^*^	
Sig. bet. periods			*p*_*2*_*=0.366*,* p*_*3*_ *= 0.302*,* p*_*4*_ *= 0.897*			
Group II *(n = 12)*						
Min. – Max.	7.0–10.0	2.0–8.0	0.0–5.0	0.0–3.0	0.0–3.0	< 0.001^*^
Mean ± SD.	8.67 ± 0.89	4.0 ± 1.86	1.33 ± 1.61	0.33 ± 0.89	0.25 ± 0.87	
* p*_*0*_		*0.121*	*< 0.001* ^*^	*< 0.001* ^*^	*< 0.001* ^*^	
* p*_*1*_			*0.020* ^*^	*0.001* ^*^	*< 0.001* ^*^	
Sig. bet. periods			*p*_*2*_*=0.272*,* p*_*3*_ *= 0.220*,* p*_*4*_ *= 0.897*			

Group I, HP + SA; Group II, CGF + SA; SD, Standard deviation; p, difference between the different studied periods, analyzed by Friedman test; *p*_*0*_, difference between Pre and each follow-up period; *p*_*1*_, p value of 1week and other periods; *p*_*2*_, p value of 1month and 3months; *p*_*3*_, comparing between 1month and 6months; *p*_*4*_, difference between 3months and 6months; *: Statistically significant at *p* ≤ 0.05

### Assisted Maximum Mouth Opening (MMO)

At baseline, the MMO mean in group I was 30.83 mm, while 1 week after the first injection, there was a significant increase with a mean difference of 2.17 mm. Furthermore, after 1 month the mean difference was 4.25 mm which was found statistically significant. Likewise, a significant improvement in MMO was noted at the 3-, and 6-months follow-ups (Table 3).

**Table 3:** Comparison between the different periods according to Maximum Mouth Opening (MMO) in each group

MMO (mm)	Pre	1week	1month	3months	6months	*p*
Group I *(n = 12)*						
Min. – Max.	27.0–36.0	30.0–40.0	32.0–42.0	35.0–43.0	37.0–45.0	*< 0.001* ^*^
Mean ± SD.	30.83 ± 3.93	33.0 ± 4.18	35.08 ± 3.90	37.33 ± 3.06	39.58 ± 3.0	
* p*_*0*_		*< 0.001* ^*^	*< 0.001* ^*^	*< 0.001* ^*^	*< 0.001* ^*^	
* p*_*1*_			*< 0.001* ^*^	*< 0.001* ^*^	*< 0.001* ^*^	
*Sig. bet. periods*			*p*_*2*_<0.001^*^, *p*_*3*_ < 0.001^*^, *p*_*4*_ < 0.001^*^			
Group II *(n = 12)*						
Min. – Max.	28.0–37.0	30.0–40.0	32.0–42.0	35.0–43.0	37.0–43.0	*< 0.001* ^*^
Mean ± SD.	31.75 ± 4.0	33.50 ± 3.78	35.50 ± 3.42	37.50 ± 2.54	39.33 ± 2.19	
* p*_*0*_		*< 0.001* ^*^	*< 0.001* ^*^	*< 0.001* ^*^	*< 0.001* ^*^	
* p*_*1*_			*< 0.001* ^*^	*< 0.001* ^*^	*< 0.001* ^*^	
*Sig. bet. periods*			*p*_*2*_<0.001^*^, *p*_*3*_ < 0.001^*^, *p*_*4*_ < 0.001^*^			

Group I, HP + SA; Group II, CGF + SA; SD, Standard deviation; p, difference between the different studied periods, analyzed by ANOVA with repeated measures test; *p*_*0*_, difference between Pre and each follow-up period; *p*_*1*_, difference between 1week and other periods; *p*_*2*_, difference between 1month and 3months; *p*_*3*_, comparing between 1month and 6months; *p*_*4*_, difference between 3months and 6months; *: Statistically significant at *p* ≤ 0.05

Pre-treatment, the MMO mean in group II was 31.75 mm, while 7 days after the injection it was significantly improved to be 33.50 mm. Similarly, a significant positive effect was found in the 1 month, and 3 months follow-up periods. By 6-month, a statistically significant increase in the MMO was found with a mean 39.33 mm (Table 2). Although both interventions showed significant improvement in MMO; the difference between the 2 groups was found to be insignificant (*p* > 0.05).

### Biochemical analysis

**Fig. 6:**
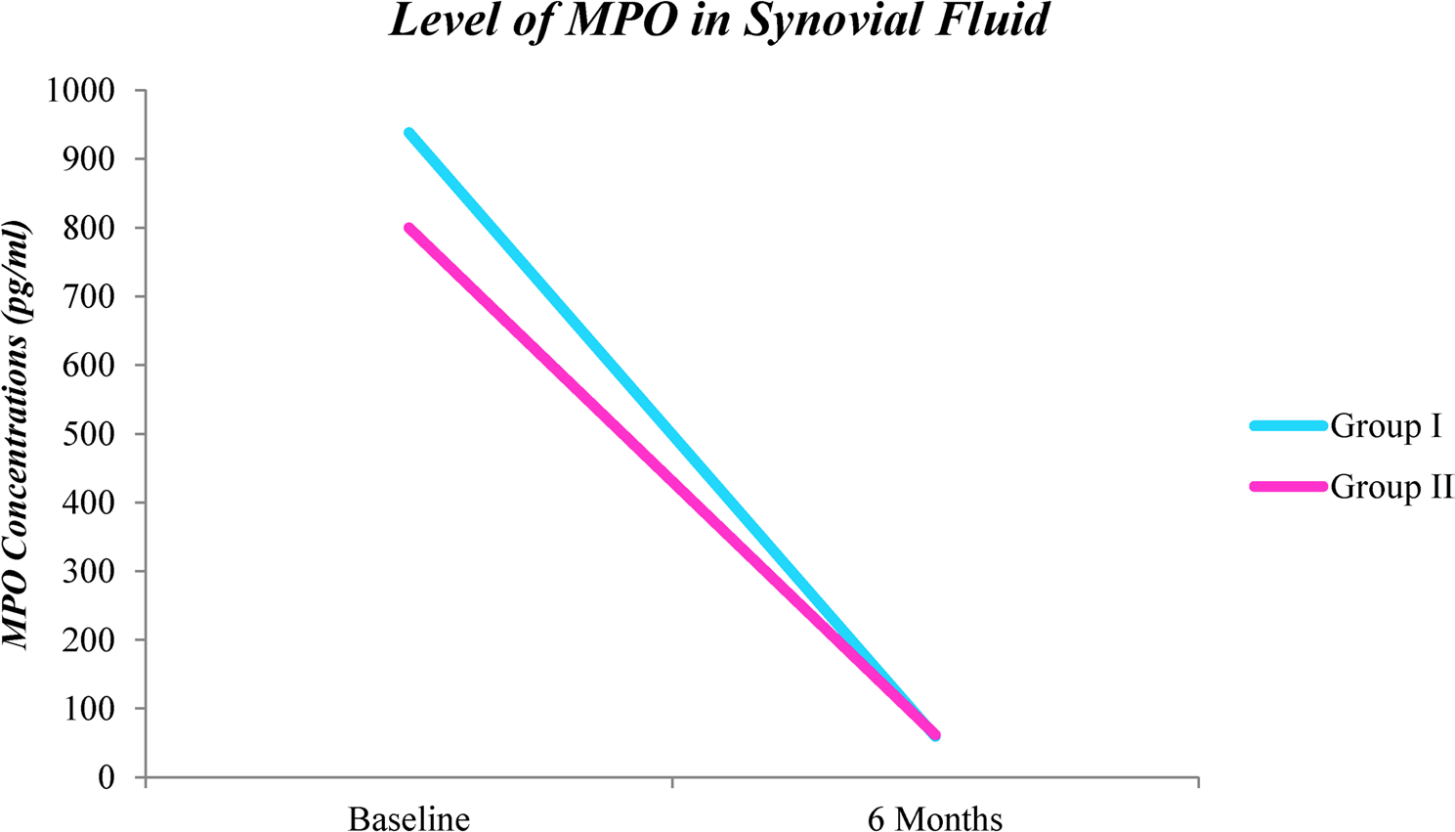
Showing change in MPO concentrations in the synovial fluids in both groups, measured by ELISA (*P* < 0.05) (group I: HDP + SA; group II: LPCGF + SA.)

At baseline, MPO levels in the SF of both groups were found to be 938.3 pg/ml, and 799.8 pg/ml respectively with no significant difference. By 6-months from the intra-articular injection, the MPO levels were reduced in both groups to reach 59.6 pg/ml, and 61.9 pg/ml respectively (*p* = 0.002). Although the MPO concentrations in the SF were significantly higher than that found after injection, there was a statistically insignificant difference between groups (Fig. 6).

Furthermore, a negative correlation was found between MPO and MMO while a positive correlation was noted between MPO and VAS (Table 4).

**Table 4:** Spearman correlation between MPO and both MMO and VAS scores

		*MMO*	*VAS*
		*Rho (p value)*	
Group I *(* *n* *= 12)*	***Pre***	-0.13 (0.68)	0.50 (0.10)
	***6 Months***	-0.18 (0.58)	0.40 (0.20)
	***Reduction***	-0.23 (0.47)	0.47 (0.13)
Group II *(* *n* *= 12)*	***Pre***	0.06 (0.86)	0.02 (0.94)
	***6 Months***	0.33 (0.30)	0.48 (0.11)
	***Reduction***	-0.51 (0.09)	-0.23 (0.47)
Total	***Pre***	-0.08 (0.71)	0.26 (0.21)
	***6 Months***	0.06 (0.78)	0.44 (0.03*)
	***Reduction***	-0.45 (0.03*)	0.06 (0.77)

Group I, HP + SA; Group II, CGF + SA; the correlation coefficient (rho) and corresponding *p*-values were calculated

## Discussion

Several studies [23–26] have attempted to assess the effect of 12.5% HD prolotherapy on the management of TMJ-ID. Their promising results have rendered this type of prolotherapy one of the effective treatment modalities for DDwoR. However, to our knowledge, studies on the effect of CGF in the management of DDwoR in literature are scarce.

Our current prospective parallel RCT was done to assess the effect of CGF in comparison to that of 12.5% HD regenerative injection in conjunction with a stabilization occlusal appliance in the management of DDwoR. With regard to our null hypothesis that there is no statistically significant difference between the effectiveness of intra-articular injection of HDPT in conjunction with a SA and that of CGF in conjunction with a SA in management of DDwoR, this hypothesis was accepted. Our findings showed that both treatment protocols have proved to be effective without significant differences between them. Nonetheless, CGF preparation needs special equipment while dextrose is easily prepared and injected. Moreover, a single injection of CGF proved to be effective, while more than one injection may be needed in HDPT.

Although no gender preference was implemented in our study; 100% of the patients were females. This goes in accordance with the studies showing that the odds for presenting TMDs were higher in females, which might be owed to hormonal influences, social factors, and higher levels of stress for females, variations in pain threshold with health-seeking attitude [41–45].

Several designs of OA can be used for management of DDwoR; however, SA is considered the appliance of choice for treating patients with DDwoR [46]. Moreover, SA relief the excessive load on the TMJ that occurs on the glenoid fossa and the head of the condyle [47]. Therefore, hard maxillary SA was used in the current study.

Reported in previous studies, post-treatment complications such as pericapsular fibrosis with further limitation in mouth opening are associated with extra-articular injection of HD in retro-discal tissues. Furthermore, the superior joint space is wider than the inferior joint space and the volume of synovial fluid is approximately 1.2 mL while the inferior joint contains only 0.9 mL. Moreover, there is no consensus in the literature on the advantage of intra-articular injections in the inferior over superior space [48]. So, an intra-articular injection of HD in superior joint space was applied [23–26].

Owing to the promising advantages of US-guided injections as improving the accuracy and efficiency of joint injections, all intra-articular injections were done under US-guidance ensuring the site of injection [49, 50].

In our study, biochemical analysis was done to detect MPO in the SF of the TMJ and evaluate the outcome of the interventions applied on a molecular basis as well. An ELISA kit designed for the quantitative measurement of MPO was used to provide an insight on the enzyme activity inside the TMJ owing to its high specificity and sensitivity [5–7].

By assessing the data of VAS, a significant relief of pain was noted in both groups in which 91.7% of the patients were pain-free. In the HDPT group, after 1 month, 2 patients complained from the return of TMJ pain after it was relieved one week post injection. By this time, those patients only received one dextrose injection. However, after 3 months where 2 consecutive dextrose injections were given, they reported relief of pain. This goes in agreement with Dasukil et al. [23] and Louw et al. [24] suggesting that multiple dextrose injections might be needed.

In group I, the MMO in 50% of the patients remained less than 40 mm after 6 months while group II showed similar limitation in 41.7% of cases; however, MMO was significantly increased from their original condition. Although the mouth opening was still considered limited in those patients, they reported pain-free mouth opening which had a clinical significance as well. These findings go in agreement with authors [23–26] who documented the effectiveness of dextrose phototherapy in reducing TMJ pain and improvement of MMO. Moreover, the results from group II go in accordance with Torul et al. [35], Yang et al. [37], and Al-Delayme et al. [49] who described similar effects of PC derivatives on TMD symptoms and MMO.

The increase in MMO could be attributed to the relief of pain, the regain of the elasticity within the articular disc that makes it foldable not interfering with the condylar translation besides the added positive effects of the SA in the treatment [51, 52]. Nevertheless, the actual rationale for the improvement remains unclear. It is of importance to mention that, on a molecular level, the decrease in inflammatory byproducts as MPO enzyme after prolotherapy, detected in our study, might reflect the healing process inside the joint which was echoed clinically as relief of TMJ pain and increase in MMO. However, further studies are needed in this field.

Although two different types of prolotherapy were investigated in the present study, both HD and CGF showed enhancement in the clinical parameters in both groups. This may be due to that regenerative injections in the joint spaces improve the micro-environment within the joint leading to the relief of the TMD symptoms in a short period as well as prevention of further progression of the TMJ-ID condition. Moreover, prolotherapy inhibits inflammatory biomarkers, produces more collagen fibrils, widens the joint spaces, and shifts the disc mobility range owing to the in-situ growth factors [23–33].

As to the results obtained from our biochemical assay, they highlight the theory that inflammatory biomarkers detected in the SF-TMJ are associated with the pathogenesis of TMJ-ID [4–7]. This goes in agreement with Arinci et al. [7] who observed an MPO activity in patients with DDwoR. Furthermore, the decrease in the MPO enzyme activity post treatment, noted in our study, may give an insight on the healing mechanism of prolotherapy. Moreover, this decrease in the enzyme activity reflects the subsiding of inflammation inside the joint which in turn reflects positively on the synovial fluid viscosity leading to lubrication and nourishment of TMJ structures. That was observed clinically by the relief of pain and the increase in MMO.

According to the results of our current RCT, the injection of 12.5% HD with a SA or the injection of CGF with a SA could be considered valuable treatment protocols in managing patients suffering from DDwoR eliminating the need for any invasive surgical interventions. In addition, the decrease in MPO enzyme in the SF of the TMJ may prevent the aggravation of the condition and promote healing of tissues.

To the best of the authors’ knowledge, this clinical trial is the first RCT to assess the effectiveness of the injection of CGF in the management of DDwoR and compare it to that of HD. However, there are limitations of our study owing to the 100% of the patients being females although no gender preference was implemented, small sample size and the lack of a control group. Nevertheless, as the patients who were included in the study were refractory to conservative treatment methods, it would be unbeneficial to the patients to implement an inactive control or a placebo group. To help those patients suffering from pain and limitation in the MMO, the authors preferred to utilize the 12.5% HD as an active control group as several studies [23–26] proved its effectiveness in the management of TMJ-ID.

## Conclusion

The current preliminary study demonstrated a novel treatment protocol for patients suffering from DDwoR with the LPGCF and a CAD/CAM SA which proved to be effective. However, further studies are recommended with larger sample size, longer follow-ups, and with comparison to other treatments.

## Data Availability

All the dataset interpreted during the present RCT are obtainable from the corresponding author upon rational request.

## Abbreviations

DDwoR: Irreducible disc displacement
TMJ-ID: Temporomandibular joint Internal derangement
RCT: Randomized controlled trial
HD: Hypertonic dextrose
PRP: Platelet-rich plasma
PRF: Platelet-rich fibrin
CGF: Concentrated growth factors
LPCGF: Liquid phase concentrated growth factor
RCF: Relative centrifugal force
SA: Stabilization appliance
VAS: Visual analogue scale
MMO: Maximum mouth opening
SF: Synovial fluid
MPO: Myeloperoxidase enzyme
TMDs: Temporomandibular disorders
TMJ: Temporomandibular joint
OAs: Occlusal appliances
CAD/CAM: Computer aided designed and manufactured
PC: Platelet concentrates
MRI: Magnetic resonance imaging
DC/TMDs: Diagnostic Criteria for temporomandibular disorders
US: Ultrasound
ELISA: Enzyme-linked immunosorbent assay
